# Progress on the Studies of the Key Enzymes of Ginsenoside Biosynthesis

**DOI:** 10.3390/molecules23030589

**Published:** 2018-03-06

**Authors:** Jin-Ling Yang, Zong-Feng Hu, Ting-Ting Zhang, An-Di Gu, Ting Gong, Ping Zhu

**Affiliations:** State Key Laboratory of Bioactive Substance and Function of Natural Medicines, Key Laboratory of Biosynthesis of Natural Products of National Health and Family Planning Commission, Institute of Materia Medica, Peking Union Medical College & Chinese Academy of Medical Sciences, Beijing 100050, China; yangjl@imm.ac.cn (J.-L.Y.); huzongfeng@imm.ac.cn (Z.-F.H.); zhangtingting@imm.ac.cn (T.-T.Z.); gad@imm.ac.cn (A.-D.G.); gongting@imm.ac.cn (T.G.)

**Keywords:** ginsenoside, biosynthetic pathway, key enzymes, synthetic biology, *Panax ginseng*

## Abstract

As the main bioactive constituents of *Panax* species, ginsenosides possess a wide range of notable medicinal effects such as anti-cancer, anti-oxidative, antiaging, anti-inflammatory, anti-apoptotic and neuroprotective activities. However, the increasing medical demand for ginsenosides cannot be met due to the limited resource of *Panax* species and the low contents of ginsenosides. In recent years, biotechnological approaches have been utilized to increase the production of ginsenosides by regulating the key enzymes of ginsenoside biosynthesis, while synthetic biology strategies have been adopted to produce ginsenosides by introducing these genes into yeast. This review summarizes the latest research progress on cloning and functional characterization of key genes dedicated to the production of ginsenosides, which not only lays the foundation for their application in plant engineering, but also provides the building blocks for the production of ginsenosides by synthetic biology.

## 1. Introduction

*Panax* species—including *Panax ginseng*, *Panax quinquefolius* and *Panax notoginseng*—have been widely used as traditional herbal medicine in Asia for thousands of years [1,2]. As the main bioactive constituents of *Panax* species, ginsenosides exhibit broad pharmacological activities such as anti-cancer, anti-oxidative, antiaging, anti-inflammatory, anti-apoptotic and neuroprotective activities [3,4,5]. The ginsenoside core structure is comprised of a triterpene aglycone, which can undergo a variety of glycosylations. Ginsenosides are categorized into three groups based on their aglycones: oleanane-type pentacyclic triterpenoid saponin Ro, protopanaxadiol (PPD)-type saponins and protopanaxatriol (PPT)-type saponins. The PPD-type and PPT-type saponins are dammarane-type tetracyclic triterpenoids, which are the major types of ginsenosides and the main bioactive constituents. To date, more than 180 ginsenosides have been isolated from *Panax* species.

**Figure 1:**
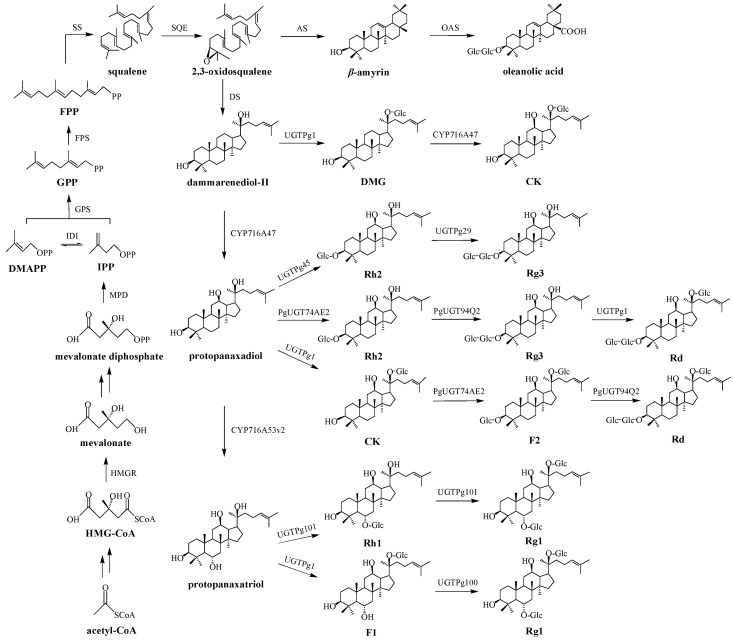
Biosynthetic pathway of ginsenosides.

The biosynthetic pathways of ginsenosides have been elucidated as shown in Figure 1 [6,7]. The whole pathway can be divided into three stages. (1) Isopentenyl diphosphate (IPP) and its isomer dimethylallyl diphosphate (DMAPP) are formed through mevalonate (MVA) pathway; (2) IPP and DMAPP are converted into 2,3-oxidosqualene; (3) Ginsenosides are produced through three reaction steps of 2,3-oxidosqualene consisting of cyclization, hydroxylation and glycosylation [8]. The biosynthetic pathways of ginsenosides comprise more than 20 steps of continuous enzymatic reactions, in which a series of key enzymes such as 3-hydroxy-3-methylglutaryl coenzyme A reductase (HMGR), farnesyl pyrophosphate synthase (FPS), squalene synthase (SS), squalene epoxidase (SQE), dammarenediol-II synthase (DS), *β*-amyrin synthase (AS), cytochrome P450 (CYP450) and UDP-glycosyltransferase (UGT) are involved.

The contents of ginsenosides in plants, cell cultures and hairy roots are too low to satisfy the increasing medical demand, and large-scale production of ginsenosides by chemical synthesis is not practical at present due to its complexity and high cost [9,10]. These problems seriously limit further pharmacological research and new drug development. In this review, we summarize recent progress on the studies of the key enzymes involved in ginsenoside biosynthesis to lay a foundation for improving the yield of ginsenosides in plants, cell cultures and hairy roots through regulation of the biosynthetic pathway and to provide building blocks for ginsenoside production by synthetic biology.

## 2. 3-Hydroxy-3-methylglutaryl Coenzyme A Reductase

3-Hydroxy-3-methylglutaryl coenzyme A reductase (HMGR) is shown to act as the first rate-limiting enzyme of the MVA pathway in plants [11]. This enzyme catalyzes the NAD(P)H-dependent reduction of HMG-CoA to mevalonate, which plays a universal role in terpene production. HMGR can regulate ginsenoside biosynthesis by regulating the production of IPP and DMAPP, which are precursors of ginsenosides. Wu et al. [12] reported the cloning and functional characterization of a full-length cDNA encoding HMGR from four-year-old *P. quinquefolius* root. The full-length cDNA named PqHMGR encodes a protein containing 589 amino acids. Bioinformatical analysis suggested that the deduced PqHMGR protein contained two transmembrane domains and one catalytic domain. PqHMGR shows high identity to HMGRs of other plants with as high as 83.8% identity to HMGR gene of *Camplotheca acuminata*. HMGR of *C. acuminata* is known as the MVA source of the terpenoid moiety that is associated with an anti-cancer monoterpenoid indole alkaloid. Therefore, PqHMGR may have an important function in terpenoid biosynthesis in *P. quinquefolius*. Expression analysis by real-time quantitative PCR indicated that PqHMGR was differentially expressed among tissues, with high expression level in the leaf and low expression level in the stem, suggesting that leaves are crucial to terpenoid biosynthesis in *P. quinquefolius*. Thus, the HMGR gene is closely related to ginsenoside biosynthesis. Luo et al. [13] reported a novel HMGR gene from *P. ginseng* (PgHMGR2). The full-length cDNA sequence of PgHMGR2 also encodes 589 amino acids with high identity (98.81%) with PqHMGR in *P. quinquefolius*. The expression level of PgHMGR2 was the highest in flower based on a real-time PCR analysis, followed by leaf and root, and the lowest in stem. Liu et al. [14] reported PnHMGR2 from *P. notoginseng* also encoded an unstable protein with 589 amino acids and possessed one catalytic domain and two transmembrane regions, which was expressed mainly in flowers followed by roots, stems, and the least in leaves. It showed 98.3% identity with *P. quinquefolius* PqHMGR. These results provide a foundation for exploring the molecular function of HMGR and the production of ginsenosides based on synthetic biology approach.

## 3. Farnesyl Pyrophosphate Synthase (FPS)

Farnesyl pyrophosphate synthase (FPS) catalyzes the conversion of geranyl diphosphate (GPP) to farnesyl diphosphate (FPP). It is a key branch-point enzyme in MVA pathway. Kim et al. [15] cloned the PgFPS gene encoding FPS from *P. ginseng* roots. The deduced amino acid sequence of PgFPS had 77%, 84%, 87% and 95% identity with FPS from *Arabidopsis thaliana*, *Hevea brasiliensis*, *Artemisia annua* and *Herba Centellae*, respectively. Southern blot analysis showed that *P. ginseng* had more than two genes encoding FPS. When the PgFPS gene was expressed in *Escherichia coli***,** the recombinant enzyme was found to possess FPS activity. Treatment of hairy roots with methyl jasmonate could increase the mRNA level of PgFPS. The accumulation of ginsenosides in root suspension cells of *P. ginseng* could also be induced by methyl jasmonate, which was related to the increased expression level of PgFPS [16]. Kim et al. [17] expressed PgFPS of *P. ginseng* in hairy roots of *Centella asiatica* and found that levels of CaDDS (*C. asiatica* dammarenediol synthase) and CaCYS (*C. asiatica* cycloartenol synthase) mRNAs were significantly improved. In particular, the upregulation of CaDDS transcripts suggests that FPS may result in alterations in triterpene biosynthesis capacity. To evaluate the contribution of FPS to triterpene biosynthesis, they treated hairy roots expressing PgFPS with methyl jasmonate and found that the content of madecassoside and asiaticoside was transiently increased by 1.15-fold. Kim et al. [18] reported that overexpression of PgFPS caused an approximate 2.4-fold increase of ginsenoside content in transgenic ginseng hairy roots. Furthermore, overexpression of PgFPS caused the upregulation of genes of ginsenoside biosynthesis, indicating a critical role of PgFPS in ginsenoside synthesis in *P. ginseng*. Wu et al. [19] cloned cDNA of FPS from *P. notoginseng* root. The FPS contains two conservative core fragments, DDIMD and QVQDDYLD, and shows 99% and 95% identity to *P. ginseng* and *C. asiatica*, respectively, with the triterpenoid structures among the above plants being very similar. These results indicate that FPS plays an important role in the triterpene biosynthesis.

## 4. Squalene Synthase

Squalene synthase (SS) catalyzes the first enzymatic step from the central isoprenoid pathway toward sterol and triterpenoid biosynthesis. It is responsible for the biosynthesis of squalene by condensing two molecules of FPP, a key precursor in phytosterol and triterpene biosynthesis. *P. ginseng* has three functional SS genes. Lee et al. [20] isolated a full-length cDNA of SS, named PgSS1, from leaf cDNA library of *P. ginseng*. The deduced amino acids of PgSS1 was 84.1%, 75.78%, 81.45% and 71.33% identical to those of *Glycine max*, *A. thaliana*, *Nicotiana tabacum* and *Oryza sativa*, respectively. The plant expression vector of the PgSS1 gene was constructed to upregulate gene expression in *P. ginseng* adventitious root. The results showed that the expression of all downstream genes including SQE and AS was upregulated, resulting in the increased levels of phytosterols and ginsenosides. These studies indicate that PgSS1 plays a regulatory role in phytosterol and ginsenoside biosynthesis. Kim et al. [21] cloned the other two homologous genes of PgSS1, named PgSS2 and PgSS3, from a *P. ginseng* expression sequence tag (EST) library. Functional complementation analysis revealed that ectopic expression of PgSS1, PgSS2 and PgSS3 in the yeast erg9 mutant strain lacking SS activity restored ergosterol prototrophy. In situ hybridization analysis showed that these three genes had different transcriptional levels in different organs of *P. ginseng*. These results indicate that expression patterns of these three SS genes are different, but they are all involved in the synthesis of squalene. Seo et al. [22] introduced the SS gene of *P. ginseng* into *Eleutherococcus senticosus* by Agrobacterium-mediated transformation. The results revealed that the increased SS activity significantly improved the production of phytosterols and triterpenoids, suggesting that SS is a key enzyme for ginsenoside biosynthesis. Jiang et al. [23] constructed a SS interference expression vector of *P. ginseng* and introduced it into *P. ginseng* callus by Agrobacterium-mediated transformation. Compared with the non-transformed callus, the expression level of SS in transformed callus decreased and the ginsenosides also had certain changes. These results indicate that SS is a key enzyme of ginsenoside biosynthesis and the yields of ginsenosides can be regulated by inhibiting SS gene expression. Jiang et al. [24] cloned SS from *P. notoginseng* and investigated its recombinant expression and preliminary enzyme activity. Bioinformatics analysis revealed that the deduced PnSS protein had a high identity of 98.07% with PgSS1 from *P. ginseng*. These observations suggest that biochemical activities of SS are essential for the production of triterpenes and phytosterols in *Panax* species.

## 5. Squalene Epoxidase

Squalene epoxidase (SQE) catalyzes the oxygenation of the double bond of squalene to produce 2,3-oxidosqualene, the first oxidation step in phytosterol and triterpenoid biosynthesis. This enzyme is another rate-limiting enzyme in this pathway. *P. ginseng* was found to have two copies of SQE genes and their sequences have low identity at the N-terminal regions. The transcripts of PgSQE1 exist abundantly in all organs, and PgSQE2 is only weakly expressed except in petioles and flower buds. Han et al. [25] cloned PgSQE1 and PgSQE2 from the leaves and adventitious roots cDNA libraries of *P. ginseng*. RNA interference of PgSQE1 in transgenic *P. ginseng* completely suppressed PgSQE1 transcription and resulted in reduction of ginsenoside production. Interestingly, silencing of PgSQE1 in RNA interference roots strongly upregulated the expression of PgSQE2 and cycloartenol synthase and resulted in enhanced phytosterol accumulation. These results indicate that PgSQE1 and PgSQE2 are regulated in different manner, and that PgSQE1 only regulates ginsenoside biosynthesis but not that of phytosterol in *P. ginseng*. Jiang et al. [26] investigated the accumulation of total ginsenosides and their monomers, and determined their relationships with the expression of SS and SQE genes in different organs of *P. quinquefolius*. They found that the expression levels of both SS and SQE were significantly different among the 14 organs and were positively correlated with accumulation of total ginsenosides and monomers. He et al. [27] cloned SQE from the root of *P. notoginseng* by PCR. The gene has 98% identity with that of *P. ginseng*. SQE of *P. notoginseng* has a FAD function domain, NAD(P)-binding Rossmann-fold domains, hydrophobicity and four transmembrane helices. This SQE may be a microsomal membrane-associated enzyme. Real time quantitative PCR analysis showed that its cDNA had different expression pattern and is highly expressed in root, especially in three-year-old root. These results reveal that SQE play an important role in the ginsenoside biosynthesis.

## 6. Dammarenediol-II Synthase and *β*-Amyrin Synthase

Oxidosqualene cyclases (OSCs) catalyze 2,3-oxidosqualene cyclization, which is a committed step in the biosynthesis of phytosterols and triterpenoids. OSCs belong to a multigene family. The cyclization of oxidosqualene can generate more than 100 kinds of triterpenoids with different skeletons. By far, the most OSC genes have been cloned from many plants. Two kinds of OSC genes involved in ginsenoside biosynthesis were isolated from *P. ginseng* and named dammarenediol-II synthase (DS) and *β*-amyrin synthase (AS). They are the key enzymes for synthesizing dammarane-type and oleanane-type ginsenosides, respectively.

Kushiro et al. [28] prepared a microsomal fraction from the hairy roots of *P. ginseng* and found that it could cyclize 2,3-oxidosqualene to dammarendiol-II in vitro. Tansakul et al. [29] designed degenerate primers according to the conserved sequence of OSC genes, and then cloned a new DS gene named PNA from hairy root cultures of *P. ginseng*. After introducing PNA into a lanosterol synthase deficient (erg7) yeast strain GIL77, dammarenediol-II was detected in the transformant, demonstrating PNA encodes DS. Based on PNA gene sequence, Han et al. [30] cloned a DS gene named DDS from the *P. ginseng* flower EST library. Their studies showed that yeast transformed by DDS gene produced dammarendiol-II and hydroxydammarenone. Methyl jasmonate can upregulate the expression of DDS gene in adventitious roots of *P. ginseng*. RNAi of DDS in transgenic *P. ginseng* resulted in silencing of DDS expression which led to reduction of ginsenoside production to 84.5% in roots. These results indicate that expression of DDS plays a vital role in the biosynthesis of ginsenosides in *P. ginseng*. Furthermore, the silencing of DDS caused increased expression of other OSCs, implying tight coordination of DDS with other OSCs because of the utilization of the same precursor. Lee et al. [31] induced the DDS gene into tobacco and produced dammarendiol-II, resulting in the increased resistance of tobacco to tobacco mosaic virus. The result also shows that DS has an important effect on ginsenoside biosynthesis. The aglycones of dammarane-type ginsenosides, PPD and PPT, are rarely detected in *P. ginseng* roots and hairy root cultures, implying that expressions of hydroxylases and glycosyltransferases are higher than that of DS, and thus dammarenediol-II formation can be considered as a rate-limiting step in ginsenoside biosynthesis. This implies that introduction and overexpression of DS in *P. ginseng* can directly lead to increased production of ginsenosides. Therefore, availability of DS gene can make it possible to engineer ginsenoside biosynthesis to improve *P. ginseng* quality, since ginseng with high dammarane-type ginsenoside content is rated as high grade. Niu et al. [32] cloned PnDS from *P. notoginseng* with high identity of 99% with DDS from *P. ginseng*, suggesting that DS is conserved across the *Panax* species. This protein contains 769 amino acids with six QW-motifs and the substrate binding DCTAE motif. Luo et al. [33] reported that *P. ginseng* DS and *P. quinquefolius* DS showed 99.5% identity, *P. quinquefolius* DS and *P. notoginseng* DS showed 99.0% identity, *P. ginseng* DS and *P. notoginseng* DS showed 98.7% identity.

AS, which catalyzes the cyclization of 2,3-oxidosqualene and produces *β*-amyrin, is a key enzyme for the synthesis of oleanane-type ginsenosides Ro. Kushiro et al. [34] cloned the cDNA sequence PNY of AS from *P. ginseng* hairy roots. After introducing PNY into yeast, *β*-amyrin was produced.

## 7. Cytochrome P450

Cytochrome P450s (CYP450s), a superfamily of monooxygenase, play critical roles in biosynthesis of plant secondary metabolites. The specific CYP450s are involved in the reactions of oxidation and hydroxylation in ginsenoside biosynthesis. Several candidate CYP450s that might be involved in ginsenoside biosynthesis have been discovered through the combination of the next generation sequencing technology and bioinformatical analysis [35]. Han et al. [36] isolated nine putative full CYP450 sequences from the EST library of *P. ginseng* adventitious roots induced by methyl jasmonate. Among these genes, the CYP716A47 gene product was selected as the putative PPD synthase because this gene was transcriptionally activated not only by methyl jasmonate treatment but also in transgenic ginseng that overexpressed squalene synthase and overproduced ginsenosides. In vitro enzymatic activity assays revealed that CYP716A47 catalyzed the oxidation of dammarenediol-II to produce PPD. Ectopic expression of CYP716A47 in recombinant WAT21 yeast yielded PPD when fed with dammarenediol-II. Furthermore, co-expression of DS and CYP716A47 genes in yeast yielded PPD without supplementing dammarenediol-II. Thus, CYP716A47 is a dammarenediol 12-hydroxylase that catalyzes the production of PPD from dammarenediol-II. This was the first report on the functional verification of CYP450 gene involved in ginsenoside biosynthesis. Chen et al. [37] analyzed the transcriptomes of *P. ginseng* and identified 133 CYP450 genes by 454 sequencing technology. Their study laid an important foundation for the further screening of CYP450 involved in ginsenoside biosynthesis. Sun et al. [38] obtained 150 CYP450s in the 454 cDNA library of *P. quinquefolius* roots, some of which encode enzymes responsible for the conversion of the ginsenoside backbone into the various ginsenosides. Finally, transcript contig00248 was selected as the CYP450 candidate most likely to be involved in ginsenoside biosynthesis through a combination of a methyl jasmonate inducibility experiment and tissue-specific expression pattern analysis based on a real-time PCR assay. Luo et al. [33] discovered the candidate CYP450 genes in *P. notoginseng*. Pn02132 and Pn00158 were most likely to be involved in hydroxylation of aglycones for gensinoside biosynthesis from 174 cytochrome P450s by phylogenetic analysis. The transcript Pn00158 was very similar to the contig00248 of *P. quinquefolius* candidate CYP450. The deduced amino acid sequence of Pn00158 had a high identity 97.95% with that of *P. ginseng* CYP716A47 which has been functionally verified. Thus Pn00158 may be CYP450 involved in ginsenoside synthesis of *P. notoginseng*. Han et al. [39] isolated CYP716A53v2 which belongs to CYP716A subfamily genes from *P. ginseng* adventitious root cDNA library. The ectopic expression of CYP716A53v2 in recombinant WAT21 yeast resulted in PPT production when PPD was added to the culture medium. In vitro enzymatic activity assays revealed that the recombinant CYP716A53v2 catalyzed the oxidation of PPD to produce PPT. The results suggested that the gene product of CYP716A53v2 was a PPD 6-hydroxylase which catalyzed the production of PPT from PPD, an important step in the formation of dammarane-type triterpene aglycones in ginsenoside biosynthesis. Dai et al. [40] engineered the synthetic pathway of PPT by introducing DS, PPD synthase, PPT synthase and NADPH-cytochrome P450 reductase from different plants in *Saccharomyces cerevisiae*. The engineered yeast strains in this work can serve as the basis for producing PPT in yeast rather than extraction from plant sources. These studies promote research on the biosynthesis pathway of ginsenosides and provide an alternative way for ginsenoside production by synthetic biology.

## 8. UDP-Glycosyltransferase

UDP-Glycosyltransferases (UGTs) catalyze the glycosylation reaction, which is the final step of ginsenoside biosynthesis. UGTs transfer glycosyl residues from activated sugars to the aglycones of ginsenosides, thus regulating properties of ginsenosides, such as bioactivity, solubility and stability. Glycosylation also determines the diversity of ginsenosides. UGTs exist in plants as a gene family with high specificity. UGTs are different by the different transferred glycosyl groups or different acceptors of glycosyl group. Chen et al. [41] isolated a kind of UGT from *P. ginseng* hairy root cultures whose molecular weight was 56.6 kD by SDS-PAGE. The enzymatic characteristics were also investigated in a preliminary study. Yue et al. [42] purified a UGT from the suspension cells of *P. notoginseng*, which can convert Rd to Rb1. This work provides the basis for further molecular study on the ginsenoside biosynthesis and it is also useful for potential application to in vitro biotransformation from ginsenoside Rd to Rb1. Chen et al. [37] identified 253 UGT genes from the transcriptomes of *P. ginseng* root by 454 sequencing technology, and then selected six transcripts of UGTs that were most likely to be involved in ginsenoside biosynthesis. Luo et al. [33] analyzed the transcriptome of *P. notoginseng* root and proposed UGT gene Pn00082 to be likely involved in glycosylation of aglycones for ginsenoside biosynthesis from 242 UGT genes by phylogenetic analysis. These studies lay an important foundation for further screening of UGT genes involved in ginsenoside biosynthesis.

In the past few years, significant advances have been made in the study of UGTs involved in ginsenoside biosynthesis. Several UGTs have been cloned and functionally identified from *Panax* species. Yan et al. [43] cloned and characterized UGTPg1 from *P. ginseng*, the first characterized UGT for glucosylation of tetracyclic triterpenoid substrates from plants which regiospecifically glycosylate C20-OH of DM and PPD. This was the key to the successful biosynthesis of CK in a one-pot reaction from simple sugars. This study provides not only a cost-effective CK-manufacturing method for its potential clinical applications, but also deepens our understanding of the biosynthetic pathways of ginsenosides within the *Panax* plants. Wang et al. [44] cloned and identified two UGTs from *P. ginseng*. UGTPg45 selectively transfers a glucose moiety to the C3-OH of PPD and its ginsenosides. UGTPg29 selectively transfers a glucose moiety to the C3 glucose of Rh2 to form a 1–2-glycosidic bond. Yeast cell factories were built to produce Rh2 and/or Rg3 from glucose based on the two UGTs and a yeast chassis to produce PPD. Jung et al. [45] sequenced and assembled the ginseng transcriptome and characterized two UGTs: PgUGT74AE2 and PgUGT94Q2. PgUGT74AE2 transfers a glucose moiety to the C3-OH of PPD and compound K to form Rh2 and F2, respectively, whereas PgUGT94Q2 transfers a glucose moiety to Rh2 and F2 to form Rg3 and Rd, respectively. Introduction of the two UGT genes into yeast together with DS and PPDS resulted in the production of Rg3. This result indicates that these two UGTs are key enzymes for the synthesis of ginsenosides, and provides a method for producing specific ginsenosides through yeast fermentation. Wei et al. [46] cloned and characterized UGT encoding genes catalyzing the glycosylation of PPT. Some key amino acid residues of the UGTs responsible for the activities and substrate regio-specificities were identified in the same study. The construction of yeast cell factories producing the bioactive ginsenosides F1 and Rh1 paves the way to manufacture these two high-value natural compounds through microbial fermentation in the future. The identity of UGTPg45 and PgUGT74AE2 was 95.4%, while the identity of UGTPg29 and PgUGT94Q2 was 99.77%. Lu et al. [47] cloned and identified a UGT gene named Pq3-O-UGT2 from *P. quinquefolius*. In vitro enzymatic assay confirmed that Pq3-O-UGT2 catalyzed the glycosylation of Rh2 and F2 to produce Rg3 and Rd. High identity of 99.32% between Pq3-O-UGT2 and PgUGT94Q2 indicated a close evolutionary relationship between *P. ginseng* and *P. quinquefolius*.

**Figure 2:**
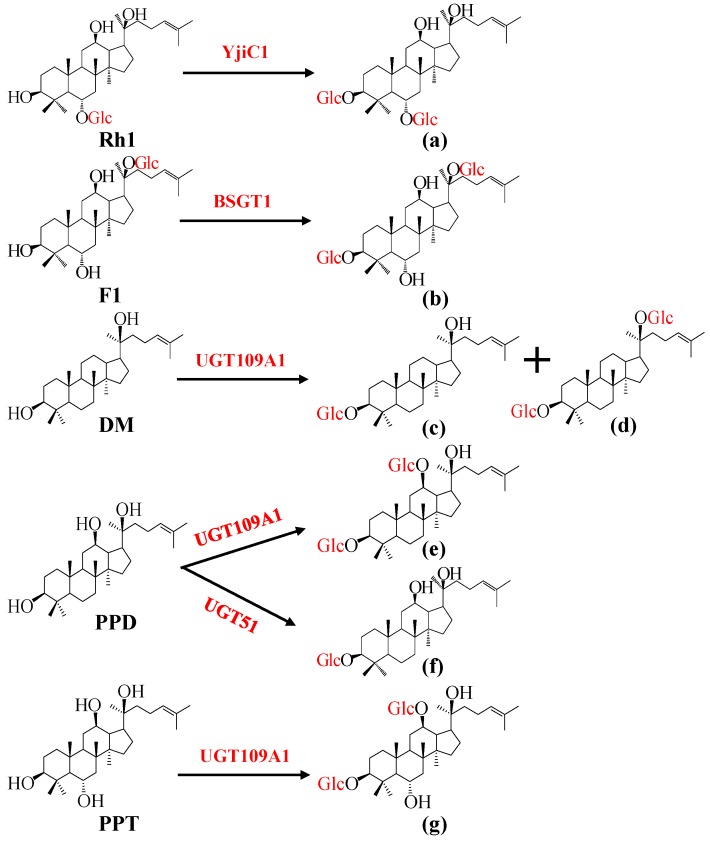
Glycosylation of ginsenosides by UGTs from microorganisms. (**a**) 3-*O*-*β*-d-glucopyranosyl-6-*O*-*β*-d-glucopyranosyl-20(*S*)-protopanaxatriol; (**b**) (20*S*)-3*β*,6*α*,12*β*,20-tetrahydroxydammar-24-ene-20-*O*-*β*-d-glucopyranosyl-3-*O*-*β*-d-glucopyranoside (ginsenoside Ia); (**c**) 3-*O*-*β*-d-glucopyranosyl-dammar-24-ene-3*β*,20*S*-diol; (**d**) 3,20-Di-*O*-*β*-d-glucopyranosyl-dammar-24-ene-3*β*,20*S*-diol; (**e**) 3,12-Di-*O*-*β*-d-glucopyranosyl-dammar-24-ene-3*β*,12*β*,20*S*-triol; (**f**) ginsenoside Rh2; (**g**) 3,12-Di-*O*-*β*-d-glucopyranosyldammar-24-ene-3*β*,6*α*,12*β*,20*S*-tetraol.

Microorganisms contain rich UGTs capable of using UDP-sugar as the sugar donor and small molecules as the sugar acceptors [48,49]. In recent years, several UGTs which can transfer glucose moieties to ginsenosides or their aglycones have been identified from microorganisms. The YjiC1 gene cloned from *Bacillus subtilis* was expressed in *E. coli* and the recombinant enzyme converted ginsenoside Rh1 into an unnatural ginsenoside 3-*O*-*β*-d-glucopyranosyl-6-*O*-*β*-d-glucopyranosyl-20(*S*)-protopanaxatriol, which is the first saponin possessing *β*-glucopyranosyl at both C3 and C6 sites (Figure 2a) [50]. Wang et al. [51] cloned an UDP-glycosyltransferase BSGT1 gene from *B. subtilis*. The recombinant BSGT1 was purified and characterized to enzymatically transform F1 into (20*S*)-3*β*,6*α*,12*β*,20-tetrahydroxydammar-24-ene-20-*O*-*β*-d-glucopyranosyl-3-*O*-*β*-d-glucopyranoside (ginsenoside Ia), which possesses an additional glucose linked into the C3 position of substrate (Figure 2b). In our study, a new UDP-glycosyltransferase UGT109A1 was also identified from *B. subtilis*. This enzyme transferred a glucose moiety to C3-OH and C20-OH of DM, and C3-OH and C12-OH of PPD and PPT respectively to produce unnatural ginsenosides 3-*O*-*β*-d-glucopyranosyl-dammar-24-ene-3*β*,20*S*-diol (Figure 2c), 3,20-Di-*O*-*β*-d-glucopyranosyl-dammar-24-ene-3*β*,20*S*-diol (Figure 2d), 3,12-Di-*O*-*β*-d-glucopyranosyl-dammar-24-ene-3*β*,12*β*,20*S*-triol (Figure 2e) and 3,12-Di-*O*-*β*-d-glucopyranosyldammar-24-ene-3*β*,6*α*,12*β*,20*S*-tetraol (Figure 2g). The metabolically engineered yeasts were then constructed to produce 3,12-Di-*O*-*β*-d-glucopyranosyl-dammar-24-ene-3*β*,12*β*,20*S*-triol by introducing the gene encoding UGT109A1 into the PPD producing strain [52]. Zhuang et al. [53] cloned a promiscuous glycosyltransferase UGT51 from *S. cerevisiae*. The semi-rationally designed UGT51 presented an 1800-fold enhanced catalytic efficiency for converting PPD to ginsenoside Rh2 in vitro and then demonstrated high-level production of Rh2 in *S. cerevisiae* by synthetic biology technology (Figure 2f).

Glycosylation is the most downstream step of ginsenoside biosynthesis. It is of great significance to enrich the diversity of ginsenosides by mining more UGTs from *Panax* species or microorganisms in the future.

## 9. Conclusions

Ginsenosides are special triterpene saponins only present in *Panax* species and accumulated especially in the plant roots. Currently, the major sources of ginsenosides are extracted from ginseng roots. Because of the limited resource and the low contents of ginsenosides in the roots, cell and tissue culture methods have been extensively explored for more rapid and mass production of ginsenosides, but the productivity of ginsenosides is still relatively poor. Therefore, overproduction of ginsenosides by metabolic engineering has been an attractive strategy to improve ginsenoside yield and investigations on their biosynthetic enzymes have received a lot of attention. In recent years, studies on the biosynthetic pathway of ginsenosides have made great progress. So far, more than 30 genes of key enzymes involved in ginsenoside biosynthesis have been cloned and functionally identified from *Panax* species and microorganisms (Table 1). Biotechnological approaches have been utilized to increase the production of ginsenosides by regulating the key enzymes of ginsenoside biosynthesis. In the meantime, synthetic biology strategies have been adopted to produce ginsenosides by introducing these genes into yeast. In the future, yeast cells may provide an alternative and attractive approach for production of ginsenosides in place of traditional extraction methods. In general, this review summarizes the latest research progress on cloning and functional characterization of key genes dedicated to the production of ginsenosides, which lays a foundation not only for their application in plant engineering but also for ginsenoside production by synthetic biology.

## Figures and Tables

**Table 1:** Key enzymes involved in ginsenoside biosynthesis.

Enzyme Name	Source	Accession No.	Reference
HMG-CoA reductase (PqHMGR2)	*P. ginseng*	JX648390	[13]
HMG-CoA reductase (PqHMGR)	*P. quinquefolius*	FJ755158	[12]
HMG-CoA reductase (PnHMGR2)	*P. notoginseng*	AKP55621	[14]
farnesyl diphosphate synthase (PgFPS)	*P. ginseng*	DQ087959	[15]
farnesyl diphosphate synthase (FPS)	*P. notoginseng*	KC953034	[19]
squalene synthase (PgSS1)	*P. ginseng*	AB115496	[20]
squalene synthase (PgSS2)	*P. ginseng*	GQ468527	[21]
squalene synthase (PgSS3)	*P. ginseng*	GU183406	[21]
squalene synthase (PnSS)	*P. notoginseng*	KC524469	[24]
squalene synthase (SS)	*P. quinquefolius*	AGK62446	[23]
squalene epoxidase (PgSQE1)	*P. ginseng*	AB122078	[25]
squalene epoxidase (PgSQE2)	*P. ginseng*	FJ393274	[25]
squalene epoxidase (SQE)	*P. notoginseng*	DQ386734	[27]
dammarenediol synthase (PNA)	*P. ginseng*	AB265170	[29]
dammarenediol synthase (DDS)	*P. ginseng*	AB122080	[30]
dammarenediol synthase (PqDDS)	*P. quinquefolius*	GU997679	[33]
dammarenediol synthase (PnDDS)	*P. notoginseng*	GU997680	[33]
dammarenediol synthase (PnDDS)	*P. notoginseng*	KC953035	[32]
amyrin synthase (PNY1)	*P. ginseng*	AB009030	[34]
cytochrome P450 (PPDS)	*P. ginseng*	JN604537	[36]
cytochrome P450 (PqPPDS)	*P. quinquefolius*	JX569336	[38]
cytochrome P450 (PnPPDS)	*P. notoginseng*	GU997665	[33]
cytochrome P450 (PnPPTS)	*P. notoginseng*	GU997666	[33]
cytochrome P450 (PPTS)	*P. ginseng*	JX036031	[39]
UDP-glucosyltransferase (UGTPg1)	*P. ginseng*	AIE12479	[43]
UDP-glucosyltransferase (UGTPg45)	*P. ginseng*	AKA44586	[44]
UDP-glucosyltransferase (UGTPg29)	*P. ginseng*	AKA44579	[44]
UDP-glucosyltransferase (PgUGT74AE2)	*P. ginseng*	JX898529	[45]
UDP-glucosyltransferase (PgUGT94Q2)	*P. ginseng*	JX898530	[45]
UDP-glucosyltransferase (Pq3-O-UGT2)	*P. quinquefolius*	KR106207	[47]
UDP-glucosyltransferase (YjiC1)	*B. subtilis*	NP389104	[50]
UDP-glucosyltransferase (BSGT1)	*B. subtilis*	KU500621	[51]
UDP-glucosyltransferase (UGT109A1)	*B. subtilis*	KY952161	[52]
UDP-glucosyltransferase (UGT51)	*S. cerevisiae*	NP013290	[53]
